# Esthetic Dentistry Required

**Published:** 1904-07-15

**Authors:** W. T. M’Lean

**Affiliations:** Cincinnati, Ohio


### ESTHETIC DENTISTRY REQUIRED.

BY W. T. M'LEAN, CINCINNATI, OHIO.

Read before the Ohio State Dental Society, December, 1903, and printed from advance proofs by courtesy of the "Dental Summary."

The therapeutics of substances derived from the mineral kingdom, either in the natural state or after modification by those who are conversant with that art, has already advanced to a place of actual permanency in our specialty, but a proper classification esthetically as applied to dentistry is far from being understood by many of our profession, if what we are compelled to gaze upon daily in the mouths of many individuals indicates anything. The prejudice that exists among persons of refinement has indicated to me that a greater degree of esthetic dentistry is required, and my apology for complying with the request of your program committee, is, that I have frequently felt justified in criticising many of my confreres, owing to the fact that we are so frequently compelled to gaze with amazement upon the incising portion of the masticatory apparatus that has been embellished, if you please, with one or more all-gold substitutes.

My purpose in making this statement upon this occasion is to particularly criticise the lack of art which is noticeable in the mouths of patients almost everywhere, and for which our profession is being criticised by refined people. To me nothing can be more inartistic than a noticeable display of gold in the mouth. Our present facilities for the restoration and replacement of the natural teeth with material other than gold is nearly perfect, and why so many neglect to utilize the more esthetic and advanced methods of tooth restoration and replacement, is, to me, an enigma. Are our dental colleges to blame for this lack of art, or is it indifference upon the part of dentists? There are many paths to follow regarding the salvation and substitution of the teeth, and, as you are aware, all conditions must be noted in order to maintain a good degree of permanance without sacrificing retention of natural appearance.

Suggestions offered the dentist by patients are too often accepted, and incongruities galore are produced. Every effort should be made in all classes of dental operations to simulate nature, overcome irregularities and lessen artificiality to the minimum by art.

I would like to appoint each gentleman present a committee of one upon "dental esthetics," so that we might make a concerted effort to eliminate at every opportunity the lack of art displayed in much dental practice. There is no reasonable excuse for placing an all-gold crown, or using the same for an abutment for bridge-work, upon any of the six anterior teeth, and rarely upon the first bicuspid. I am fearful of the degradation our profession shall experience erelong, if there is not a change made in this particular lack of refinement in rendering dental service. It has been said by some that patients insist upon having certain things done, and if you do not do some incongruous operation upon their teeth as they specially request, they will go elsewhere. When patients of that class consults me, I set them right in a very few minutes, by politely offering them the opportunity of doing their work themselves if they know so much about it, and proceed to dexterously dismiss them from my office.

Gentlemen, this is a topic of greater interest to dentistry than some of you at this time may appreciate, and I have no objection to being placed on record as stating that it is to me the most barbarous practice possible, and should be eliminated from all esthetic dental practice. If this condition is not remedied our profession will be placed among the tonsorial and medicated-bath class of business enterprises. Esthetic dental service will always be appreciated by the elite, and a few practitioners will attract that class of patronage. But why not all be honored likewise? Many practitioners are seemingly indifferent to the more artistic methods which may be employed. The criticism most likely to be made of the views herewith expressed is the lack of stability where gold is not used. I wish to state that the fracture of porcelain when used in place of gold usually results from imperfect adaptation or mal-occlusion and not from any weakness of the material mentioned.

In concluding, I most earnestly hope that esthetics, as applied to dentistry, may be more carefully considered, so that our profession will not be degraded among other professions and societies of art.

### DISCUSSION.

DR. W. A. BARBER: I find it a difficult matter to discuss a paper when I so heartily agree with the essayist. I do not think any one of us can have any difference of opinion regarding the lack of art displayed in placing an all-gold crown on teeth referred to by the Doctor, but with one or two points in the paper I cannot agree.

I cannot help believing that a reaction has set in against the use of the gold crown on anterior teeth, not only among the dentists but among our patients as well. For a time every one seemed to be carried away with the idea that they must have an all-gold crown conspicuously displayed in their mouths, and I firmly believe we do not have to contend with this state of affairs as much as heretofore. Is it not more often the case when you are about to insert a large gold filling in an anterior tooth that your patient will say, "Doctor, can you not use something else? I dislike a display of gold so much." Does it not prove something is taking place in the minds of the laity against the use of this method.

Again I differ with the Doctor in regard to the handling of that patient who wants that incongruous operation performed. He informs us that he dismisses them from his office. I cannot consider that the right kind of an impression to instill into the mind of the young practitioner, it is contrary to his high idea of professional treatment. That young man's rent and a dozen other expenses may be staring him in the face, and he should be instructed how to handle that patient without a financial loss to himself, rendering that patient the best possible services known to modern dentistry and at the same time retaining his self-respect.

American dentists lead the world, and the reason given by eminent men both at home and abroad is the fact that American dentists are teachers, and I do not consider that teaching is confined to the halls of our universities.

The dentist at his chair is teaching day after day and year after year, and he should be able to satisfy his patients that what he wishes to do for them is just what he would have done in his own mouth, the same conditions present.

Dr. L. E. Custer, Dayton: The first thing that I would commend is the brevity of the paper, and being directly to the point. I believe, however, that the time is fast arriving when the dentist will be better able to comply with the requirements of the paper. The use of porcelain in many branches of dentistry is certainly increasing, and whenever porcelain is used for the displacement of any metal I feel that the operation is at least more esthetic; whether it shall be more durable or not is the question to be determined by time.

Dr. Stephen, Cleveland: The paper brought out the point that we must dispense with the glare of gold. It did not exactly state just what we must use. An esthetic dentist should use porcelain in the anterior teeth, I believe was the essayist's contention. Porcelain is not a practical substance, where these crowns are placed, as it is liable to fracture and yet I have put these on for years. I fill with the porcelain and yet the porcelain will break off at times. Now, if we are going to put on porcelain crowns we must have something that, when it breaks, we can replace comparatively easily.

I was glad to see that the use of certain porcelain crowns that could be replaced—Davis crowns for example—are advocated. While I am not connected in any way with the use or sale of that crown, still this crown has its claims for easy replacement when broken, and is going to bring porcelain into use in bridge-work, as well as crown-work; but they will have to modify the crown a little, and if there is any gentleman present who is able to bring that about I think we can make it very useful to the profession.

Dr. W. T. McLean: It is the downfall of our profession, just as sure as fate, to use indiscriminately gold caps. How many laterals and centrals do we see in people's mouths? Some dentists put sixteen or eighteen metal crowns in a single mouth for a small sum. Because it is easier to put them in than to fill the teeth properly. It is not the dental college that is graduating men who have no more sense of art, or education than that. I tell you, gentleman, the dental profession twenty years hence will be so far advanced in esthetics, that we shall see fewer gold crowns. I believe this from the education that is being given our dentists at the present time.

